# Multiple Breath Nitrogen Washout: A Feasible Alternative to Mass Spectrometry

**DOI:** 10.1371/journal.pone.0056868

**Published:** 2013-02-15

**Authors:** Renee Jensen, Sanja Stanojevic, Karyn Gibney, Juliana Giraldo Salazar, Per Gustafsson, Padmaja Subbarao, Felix Ratjen

**Affiliations:** 1 Division of Respiratory Medicine, Department of Pediatrics, the Hospital for Sick Children, University of Toronto, Toronto, Canada; 2 Child Health Evaluative Sciences, Research Institute, the Hospital for Sick Children, University of Toronto, Toronto, Canada; 3 Department of Pediatrics, Central Hospital, Skövde, Sweden; University of Tübingen, Germany

## Abstract

**Background:**

The lung clearance index (LCI), measured by multiple breath washout (MBW), reflects global ventilation inhomogeneity and is a sensitive marker of early cystic fibrosis (CF) lung disease. Current evidence is based on a customized mass spectrometry system that uses sulfur hexafluoride (SF_6_) as a tracer gas, which is not widely available. Nitrogen (N_2_) washout may be better suited for clinical use and multi-center trials.

**Objective:**

To compare the results obtained from a N_2_ washout system to those generated by the SF_6_ based system in healthy children and children with CF.

**Methods:**

Children with CF were recruited from outpatient clinics; healthy children were recruited from the Research4Kids online portal. Participants performed MBW_SF6_ (Amis 2000, Innovision, Denmark) and MBW_N2_ (ExhalyzerD, EcoMedics, Switzerland) in triplicate, in random order on the same day. Agreement between systems was assessed by Bland-Altman plot.

**Results:**

Sixty-two healthy and 61 children with CF completed measurements on both systems. In health there was good agreement between systems (limits of agreement −0.7 to 1.9); on average N_2_ produced higher values of LCI (mean difference 0.58 (95% CI 0.42 to 0.74)). In CF the difference between systems was double that in health with a clear bias towards disproportionately higher LCI_N2_ compared to LCI_SF6_ at higher mean values of LCI.

**Conclusion:**

LCI_N2_ and LCI_SF6_ have similar discriminative power and intra-session repeatability but are not interchangeable. MBW_N2_ offers a valid new tool to investigate early obstructive lung disease in CF, but requires independent normative values.

## Introduction

Pathologic changes associated with cystic fibrosis (CF) lung disease occur in early childhood, but have historically gone undetected until the onset of clinical symptoms, at which point irreversible lung damage may have already occurred [1]. Consequently, over the last ten years the focus of clinical care in CF has shifted to early intervention and prevention of these structural changes. To facilitate early intervention there is a pressing need for surrogate markers of early obstructive lung disease that are also sensitive enough to detect treatment effects. [2]

Spirometric measures, such as forced expired volume in one second (FEV_1_), have traditionally been used in the assessment of CF lung disease due to their direct correlation with morbidity and mortality.[3] However, FEV_1_ tends to remain within normal limits in a high percentage of children, despite radiographic evidence of airway damage. [4], [5], [6], [7] This is likely due to the fact that these measures are primarily influenced by resistive changes in the large airways and thus not reflective of the patchy distribution of small airway pathology characteristic of early CF lung disease. [8] In addition to this inherent insensitivity, young children are also often not developmentally advanced enough to perform complicated respiratory maneuvers. The lung clearance index (LCI), as measured by multiple breath washout (MBW), reflects global ventilation inhomogeneity (VI) and as such is a highly sensitive marker of early obstructive lung disease.[9], [10], [11] Furthermore, LCI is more sensitive than other measures of lung function in detecting structural changes identified by high resolution computed tomography (HRCT) imaging [4], [6], [7]. MBW is performed during tidal breathing and requires only passive co-operation, it is therefore feasible during infancy and early childhood. Importantly, LCI tracks from preschool to school-age and has been found to precede subsequent abnormalities in spirometric indices [12].

To date most evidence for LCI has been collected using mass spectrometry based MBW systems. [9], [10], [11] The equipment is immobile, expensive and uses sulfur hexafluoride (SF_6_) as its inert tracer gas. Therefore, the current customized system is neither suitable for multi-center clinical research nor clinical practice. Multiple breath nitrogen washout (MBW_N2_) offers a possible alternative to mass spectrometry based SF_6_ washout (MBW_SF6)_. N_2_ is a resident gas and permeates even poorly ventilated lung units, which may not be the case during MBW_SF6_. Thus, the physiological attributes of the respective tracer gases may lead to differences in measurements obtained with the two systems. The aim of this study was to determine whether the results of MBW_N2_ and MBW_SF6_ can be used interchangeably in both healthy children and children with CF. In addition, we aimed to quantify the discriminatory power of LCI, as measured by MBW_N2_ and MBW_SF6_, to differentiate health and disease throughout a range of pulmonary function abnormalities in CF.

## Methods

This study was approved by the research ethics board (REB) at the Hospital for Sick Children (HSC), Toronto, Canada (REB# 1000019945). Informed written consent was obtained from the parents or guardians of healthy children and children with CF. Assent was obtained from subjects when appropriate.

### Study Subjects

Families with eligible children between the ages of 3 and 18 years attending a routine visit to the CF outpatient clinic of the HSC were invited to participate in our study. Eligibility was defined as a diagnosis of CF by a positive newborn screening test or at least one clinical feature of CF in combination with either a documented sweat chloride ≥60 mEq/L by quantitative pilocarpine iontophoresis or a genotype with two CF-causing mutations. Children with acute respiratory symptoms, inter-current respiratory infections, or chronic lung disease not related to CF were excluded from participation; as were patients requiring supplemental oxygen.

Healthy controls were recruited from siblings of children attending our Respiratory Medicine outpatient clinics, children of staff members and through the Research4Kids online portal supported by the SickKids Research Institute. Health was defined as no history of chronic use of bronchodilator or controller medication for asthma symptoms, no chronic lung disease and no active or passive exposure to cigarette smoke. All subjects were free of acute respiratory tract symptoms for at least four weeks prior to testing. Children with any history of wheeze within the previous two years were excluded from the study.

Participants performed MBW_SF6_ and MBW_N2_ in triplicate, in random order on the same day. All children attempted to perform spirometry, while plethysmographic lung volume measurement was attempted by children age seven and older. Lung function testing was performed according to American Thoracic Society (ATS) standards using the Vmax system (VIASYS CareFusion San Diego, California, USA). [13], [14] Children between the ages of 3 and 6 years performed spirometry to ATS ERS standards for pre-school lung function testing [15] using the Easy-on-PC system (ndd, Zurich, Switzerland). Height, weight, BMI and spirometry outcomes were standardized for age, body size and sex.[16], [17], [18]

### MBW Testing

#### MBW_SF6_

A mass spectrometer (AMIS 2000; Innovision A/S, Odense, Denmark) based set up and technique was used to perform MBW testing with a SF_6_/He gas mixture as previously described.[9], [10], [11] Briefly, subjects breathed a gas mixture containing 4% SF_6_, 4% He, 21% O_2_, balance N_2_ via an open circuit bias flow system through either a mask or mouthpiece and an attached heated pneumotachograph (3700 series Hans Rudolph, Shawnee, KS, USA) which measures flow by pressure differential, until equilibrium was reached. Once the inert tracer gas (SF_6_) stabilized at 4%, the gas source was removed during the start of exhalation and the subject breathed room air until end-tidal SF_6_ concentration reached below 1/40^th^ of its starting concentration for at least three breaths. Depending on individual feasibility, either a mask (Silkomed, Rendell Baker Masks size 3, Rusch Canada Inc., Benson Medical Industries, Markham, Ontario) filled with therapeutic putty (Air Putty, Sammons Preston Canada Inc., Mississauga, Ontario) or mouthpiece (VacuMed model #1004, Ventura, CA, USA) with nose clips was used. All subjects used the same size pneumotachograph with a total post gas sampling point dead space of 15.4 ml; pre-gas sampling point dead space was considered to be zero for mouthpiece and 10 mls for mask and putty [19]. Calculation of signal delay and subsequent alignment of flow and gas concentration signals with appropriate BTPS correction was performed as previously described. [9], [10], [11]

#### MBW_N2_

MBW_N2_ was performed using an open circuit, bias flow system (Exhalyzer D^®^, EcoMedics AG, and Duernten, Switzerland) and associated software (Spiroware^®^ 3.1 EcoMedics AG). This MBW_N2_ device uses an indirect technique to determine N_2_ concentration. Oxygen (O_2_) and carbon dioxide (CO_2)_ were measured during testing; N_2_ was then calculated based on Dalton's law of partial pressures.[20] CO_2_ was measured using a mainstream infrared CO_2_ sensor (Capnostat^®^ 5, Respironics Novametrix LLC, Wallingford CT, USA). Incorporated into the CO_2_ sensor was a sampling port where O_2_ was measured side stream at a rate of approximately 3 ml/s to an internal O_2_ analyzer (Oxigraf Inc, Mountain View, CA, USA). Flow was measured by an ultrasonic flow head [21] inline along the breathing circuit, and volume was derived from the flow signal by integration. Due to differences in O_2_ and CO_2_ sensor response times a speeding algorithm was applied to the O_2_ signal to reduce the response time to approximately 110 ms in order to align gas signals. Synchronized gas signals were time-shifted to align with flow as described by Singer et al, 2012.[20]

In contrast to MBW_SF6_, a wash-in phase using a test gas was not required. The subject breathed 100% O_2_ during wash out to reduce the concentration of N_2_ in the lungs to below 1/40^th^ of the starting concentration. The switch from room air to 100% O_2_ was automated, eliminating the need for manual disconnect as was done during MBW_SF6_. As there was no parallel wash-in phase during MBW_N2_ subjects were allowed to re-equilibrate in room air between test trials. Time between trials was at minimum the time required to washout on the previous trial.

### Offline Data Analysis

Synchronized data files from both systems were analyzed by trained observers using custom written analysis software (TestPoint, Capital Equipment Corp., Billerica, MA, USA). To assess inter-observer variability of offline MBW results, the N_2_ data files from 40 subjects (20 HC and 20 CF) were independently over-read by two observers. Quality control standards, as proposed by the ERS working group [19], were used as guidelines for technical acceptability during offline data analysis.

### Indices calculated

Functional residual capacity (FRC) is calculated by dividing the net amount of inert tracer gas exhaled over the course of the washout by the difference in end-tidal marker gas concentration (Cet) from the beginning to the end of washout. [22] LCI represents the number of FRC turnovers required to reduce the end-tidal concentration of tracer gas to 1/40^th^ of the starting concentration and is calculated by dividing the sum of exhaled tidal breaths (cumulative exhaled volume (CEV)) by simultaneously measured FRC. [22]

### Statistical Analysis

For each outcome, agreement between the SF_6_ and N_2_ systems was assessed using Bland-Altman plots. [23] A t-test was used to test whether MBW outcomes in healthy controls were different from children with CF. Additional analysis used simple linear regression to determine whether the differences between the two systems could be explained by body size and/or lung function. A p-value <0.05 was regarded as statistically significant.

## Results

**Figure 1:**
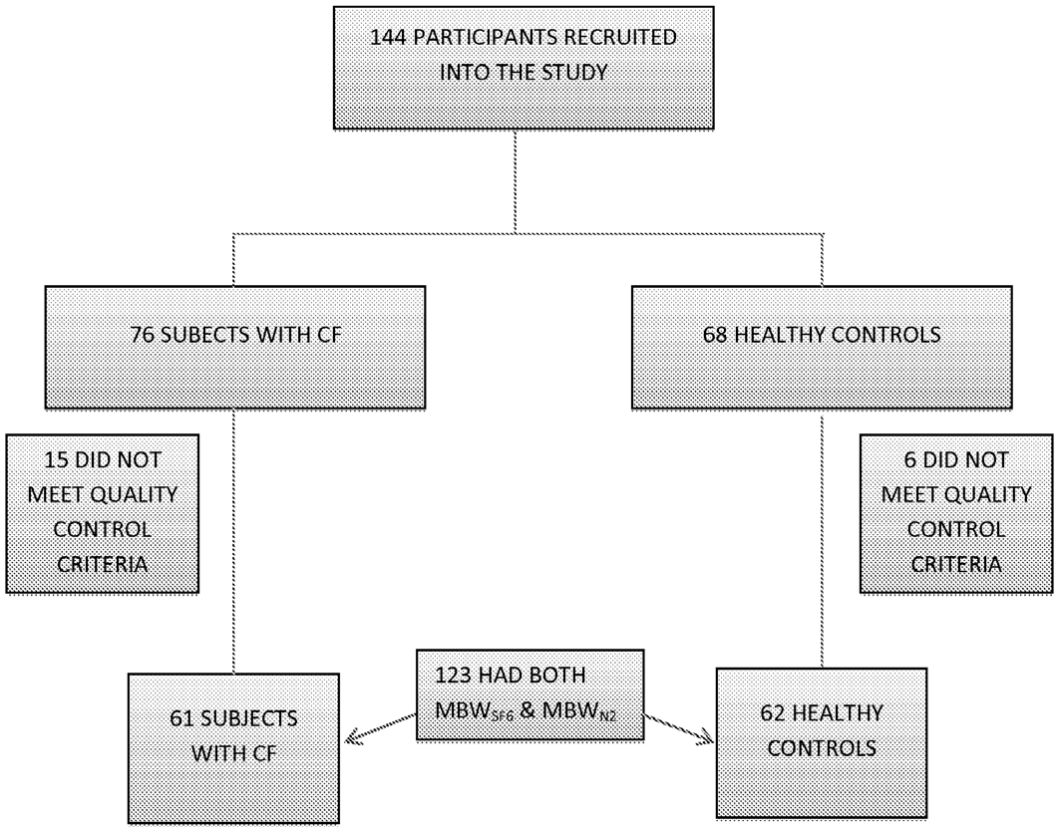
Study Participant Flow Diagram.

144 children (68 healthy controls and 76 CF) were enrolled into this study (Figure 1). Subjects who failed to meet MBW_SF6_ and or MBW_N2_ quality control criteria were excluded from analysis (Figure 1). In most cases, subjects failed to meet quality control criteria due to inability to maintain stable breathing pattern, leak around interface, or incomplete washout. In total 62 HC (91%) and 61 CF (80%) had paired measurements on both systems available for analysis. Both groups were well matched for age and sex. As expected the healthy group were taller and heavier than CF subjects (Table 1). Spirometry (FEV_1_ z-scores) was reduced in the CF group compared to healthy controls, whereas FRC measured by plethysmography (percent predicted) was elevated in CF compared to healthy controls (Table 1). Each subject completed at least two acceptable MBW trials. Overall the within test occasion variability (coefficient of variation (CV) of all trials) was similar for both systems, and similar in health and disease (Table 2). There was no evidence that the CV was affected by increased ventilation inhomogeneity as CV was constant across the range of LCI.

**Table 1:** Characteristics of the study population (presented as mean (SD) unless otherwise indicated).

	CF n = 61	Health n = 62
**% Females**	41%	39%
**Age (years)** mean (range)	11.0 (3–17)	10.9 (3–18)
**Weight (kg)** Centile-for-age	45.7 (27.7)	69.0 (22.4)
**BMI** Centile-for-age	46.5 (25.3)	57.2 (27.7)
**Height (cm)** Centile-for-age	47.6 (29.5)	75.8 (21.1)
* **FRC_pleth_ (% pred)**	118.8 (19.9)	105.5 (14.6)
** **FEV1 (Z-score)**	−1.2 (1.5)	−0.2 (0.8)
** **FEV1 (% pred)**	85.9 (18.2)	97.8 (10.2)

* FRCpleth measurements were obtained in n = 44 HC and n = 30 CF.

** FEV_1_ measured in n = 53 HC and n = 56 CF.

**Table 2:** Summary of MBW outcomes (presented as mean (CV) unless otherwise indicated).

	HC mean (CV)	CF mean (CV)	P-value
**Sample Size**	61	62	
**LCI_SF6_**	6.19 (0.05)	10.05 (0.05)	<0.001
**LCI_N2_**	6.81 (0.05)	11.29 (0.05)	<0.001
**FRC_SF6_ (L)**	1.60 (0.06)	1.41 (0.06)	0.185
**FRC_N2_ (L)**	1.92 (0.07)	1.89 (0.05)	0.948
* **FRC_pleth_ (L)**	2.25 (0.79)	2.31(0.97)	0.471

* FRC_pleth_ measurements were obtained in n = 44 HC and n = 30 CF; results presented as mean (SD).

### LCI comparison between systems

**Figure 2:**
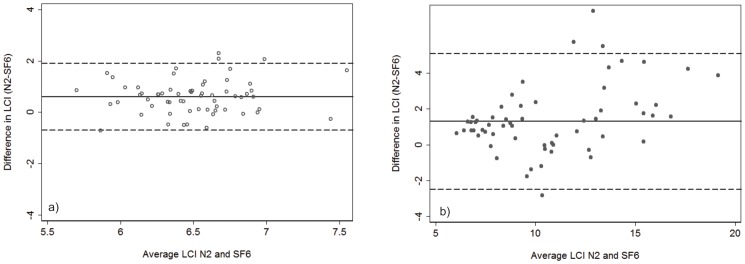
Bland Altman Plot of the agreement between LCI _N2_ and LCI_SF6_ in a) healthy controls and b) subjects with Cystic Fibrosis. The solid horizontal line represents the mean difference, and the dashed lines represent the limits of agreement (mean difference+/−2SD). In health, there was good agreement between the systems, the mean difference (LCI_N2_−LCI_SF6_ was 0.61 (95% CI 0.45 to 0.78), limits of agreement (−0.7 to 1.9)); whereas in CF there was an obvious bias (mean difference = 1.41 (95% CI 0.92 to 1.90), limits of agreement (−2.4 to 5.2)) such that LCI_N2_ increased disproportionately to LCI_SF6_ as mean LCI increased.

In both systems LCI identified the same proportion (96%) and the same subjects as abnormal. On average, in healthy subjects MBW_N2_ generated higher values of LCI (mean difference (LCI_N2_−LCI_SF6_) = 0.61 (95% CI 0.45 to 0.78), but there was good agreement between systems with uniform scatter around the mean difference (limits of agreement −0.7 to 1.9) (Figure 2a). In CF, the mean difference between systems (LCI_N2_−LCI_SF6_) was double that in health (1.41 (95% CI 0.92 to 1.90), with a clear bias such that LCI_N2_ was disproportionately higher than LCI_SF6_ as the average LCI values increased (Figure 2b).

The same bias was not observed when LCI_SF6_ was compared to LCI measured using another low density gas, helium (LCI_He_). While the variability in the difference between LCI_SF6_ and LCI_He_ increased as the average LCI increased, the scatter was uniform on both sides of the mean difference (data not shown).

### FRC comparison between systems

**Figure 3:**
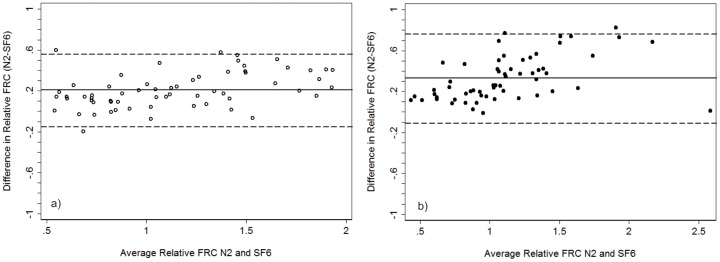
Bland Altman Plot of the agreement between FRC _N2_ and FRC_SF6_ in a) healthy controls and b) subjects with Cystic Fibrosis. The solid horizontal line represents the mean difference, and the dashed lines represent the limits of agreement (mean difference+/−2SD). FRC was crudely corrected for body size (FRC/height*100). In health N_2_ produced higher values of FRC; the mean difference (FRC_N2_−FRC_SF6_) was 0.21 (95%CI 0.16; 0.25), limits of agreement (−0.15; 0.56) with no bias observed between systems. In CF the mean difference was 0.33 (95%CI 0.27; 0.38), limits of agreement (−0.11; 0.76) with the difference between systems becoming disproportionately greater with higher adjusted FRC.

As a crude way to adjust for body size, FRC measurements from both systems were adjusted for height (FRC/height)*100 and expressed as relative FRC. In health MBW_N2_ produced higher values of FRC (mean difference (FRC_N2_−RC_SF6_) = 0.21 (95% CI 0.16; 0.25)), with no bias observed between systems (limits of agreement −0.15; 0.56) (Figure 3a). In CF the difference between the two systems was greater than in health (mean difference = 0.33 (95%CI 0.27; 0.38)), and the difference was disproportionately greater with higher average adjusted FRC (Figure 3b).

**Figure 4:**
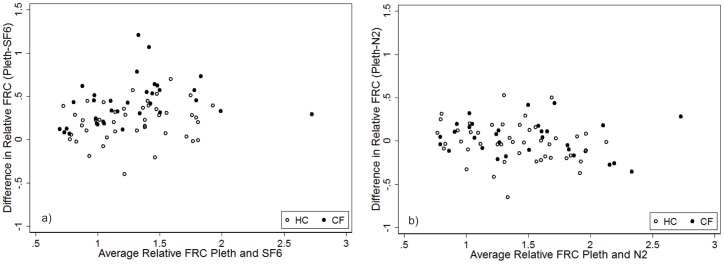
Bland Altman Plot of the agreement between a) FRC_pleth_ and FRC_SF6_ and b) FRC_pleth_ and FRC_N2_. Healthy controls are represented by the open circles, and subjects with CF by the solid circles. FRC was crudely corrected for body size (FRC/height*100). FRC_N2_ more closely agreed with FRC_pleth_ with the difference between FRC_pleth_ and FRC_SF6_ suggestive of trapped gas volume.

**Figure 5:**
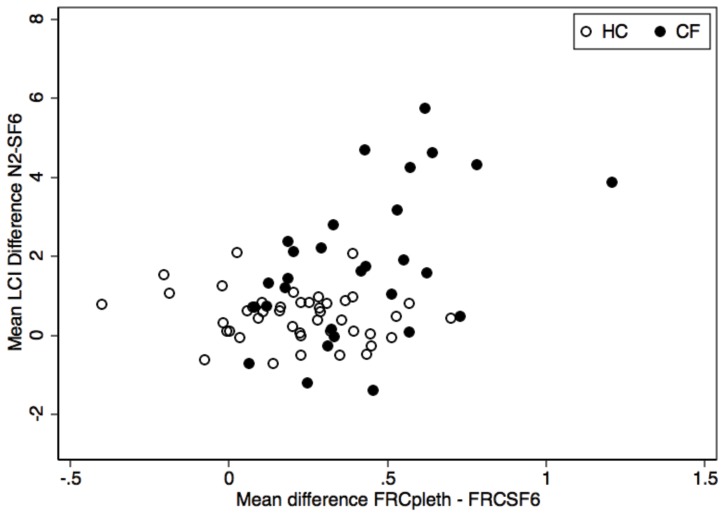
Comparison of the mean difference in LCI between systems to volume of trapped gas (FRC_pleth_−FRC_SF6_). The volume of trapped gas increased as LCI_N2_ increased disproportionately to LCI_SF6_ suggesting that the N_2_ system was measuring volume not captured during MBW_SF6_.

Thirty CF and 44 HC had measurements of all three FRC outcomes (FRC_pleth_, FRC_SF6_ and FRC_N2_) (Table 2); for comparison each FRC measure was corrected for body size in the same manner (FRC/height*100). FRC_N2_ more closely agreed with FRC_pleth_ (Figure 4). As the difference between FRC_pleth_ and FRC_SF6_ may represent the volume of gas in extremely slowly ventilated lung units, we compared the difference in LCI between systems to trapped gas volume (FRC_pleth_−FRC_SF6_). We observed that the volume of trapped gas increased as LCI_N2_ increased disproportionately to LCI_SF6_ suggesting that the N_2_ system is measuring volume not captured using SF_6_ (Figure 5).

### Additional comparisons between systems

**Figure 6:**
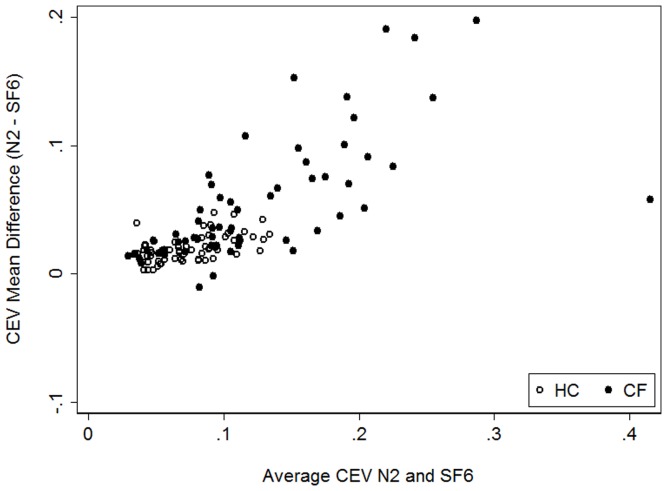
Bland Altman Plot of the agreement between CEV_N2_ and CEV_SF6_. Healthy controls are represented by the open circles, and subjects with CF by the solid diamonds. CEV was adjusted for body size (CEV/height*100). In health there was good agreement between systems, mean difference (CEV_N2_−CEV_SF6_) was 0.20 (95% CI 0.017; 0.022), limits of agreement (−0.001; 0.041) with no bias observed between systems. In CF there was a strong bias such that CEV_N2_ became disproportionately higher than CEV_SF6_ with increasing mean values of CEV (mean difference (0.054 (95% CI 0.042; 0.067), limits of agreement (−0.041; 0.150)).

As LCI is the cumulative expiratory volume (CEV) divided by FRC, we examined the agreement of CEV_N2_ and CEV_SF6_, corrected for pre and post gas sampling point dead space, between systems and found good agreement in health with no bias observed (limits of agreement −0.001 to 0.041) (Figure 6). In CF, there was a strong bias such that CEV_N2_ was disproportionately higher than CEV_SF6_ with increasing mean values of CEV (limits of agreement −0.041 to 0.150).

Since CEV is the product of tidal volume (Vt) and number of breaths required to complete washout, we compared the Vt/FRC ratio between systems. Both variables were corrected for pre and post gas sampling point dead space. While the variability of Vt/FRC was greater in health than in CF, there was minimal difference and no bias observed when the two systems were compared (data not shown). Healthy subjects required an additional 5 breaths to complete washout during MBW_N2_ compared to MBW_SF6_ (mean (SD): 35(14) vs. 30(13), p<0.001). CF subjects required an additional 18 breaths to complete washout using the N_2_ system (mean (SD): 56 (26) vs. 38(14), p<0.001). This indicates that the bias observed in CEV between systems is related to number of breaths. When the difference in breath number was compared to volume of trapped gas we found that number of breaths required to complete washout using N_2_ increases proportionally to volume of trapped gas (data not shown).

Respiratory rate was lower during MBW_N2_ compared to MBW_SF6_ in both health (17 breaths/minute vs. 19; p<0.001) and disease (18 breaths/minute vs. 21; p<0.001)), but was constant across the range of LCI; there was no bias observed in respiratory rate between the two systems (data not shown).

### Comparison between systems and disease severity

**Figure 7:**
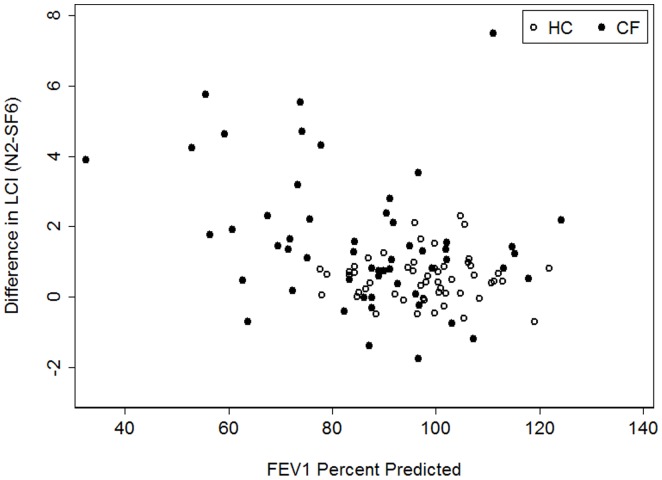
Comparison of difference in LCI (LCI_N2_−LCI_SF6_) to FEV_1_ (% predicted). Healthy controls are represented by the open circles and subjects with CF by the solid circles. The difference in LCI was greater as FEV_1_ became lower such that on average LCI_N2_ was disproportionately higher than LCI_SF6_ in subjects with abnormal spirometric findings.

To determine whether the difference in LCI between systems was related to lung function we compared the difference in LCI across a range of lung function abnormalities. The difference in LCI between the two systems was greater as lung function worsened (i.e. lower values of FEV_1_ (Figure 7) and higher values of FRC_pleth_ (data not shown)), such that on average LCI_N2_ was disproportionately higher than LCI_SF6_ in subjects with abnormal lung function compared to those with normal spirometric and plethysmographic findings (data not shown). The observed differences could not be explained by differences in age or body size (height, weight, BMI (data not shown)).

Finally, to investigate the contribution of factors explaining the observed differences in LCI between systems, a linear regression was performed for each factor separately (Table 3). Greater breath number during MBW_N2_ compared to MBW_SF6_ explained most of the variability (24%) in the difference in LCI while trapped gas and zFEV1 explained 15% and 13% of the variability respectively.

**Table 3:** Linear univariate regression analysis investigating difference in LCI between the two systems.

	R^2^
**Difference in breath number**	0.242
**zFEV_1_**	0.129
**FRC_pleth_ percent predicted**	0.097
**Difference in tidal volume**	0.001
**Trapped Gas (FRC_pleth_−FRC_SF6_)**	0.147

## Discussion

To the best of our knowledge, no other study has directly compared outcomes measured by MBW_N2_ to those measured by both MBW_SF6_ and traditional lung function tests in healthy children and children with CF. LCI_N2_ and LCI_SF6_ had similar discriminative power and intra-session repeatability but are not interchangeable as LCI_N2_ was on average higher than LCI_SF6_. As such, interpretation of parameters measured by MBW_N2_ will require independent normative values to define an appropriate upper limit of normal.

The feasibility of using MBW_N2_ in a pediatric clinical setting has recently been described but this study did not include head to head comparison to other technologies[24]. Two studies have previously compared alternative MBW systems to mass spectrometry based MBW_SF6_.[25],[26]. However, both used SF_6_ as the tracer gas and neither performed between system comparisons in the same individual nor compared MBW based lung volume measurements to plethysmographic FRC measurements; therefore results are not directly comparable to our study.

Although the LCI and FRC were comparable between systems in health, albeit higher using N_2_, the bias observed in CF subjects clearly demonstrates that the two systems cannot be used interchangeably. These observed differences could potentially be explained by differing physiological properties of SF_6_ and N_2_. SF_6_ is a heavy gas and thus may behave differently in the periphery of the lung than a lighter gas (He or N_2_); however comparison of LCI_SF6_ to LCI_He_ in CF did not demonstrate the same bias observed between LCI_SF6_ and LCI_N2_. The endogenous nature of N_2_ results in the contribution of gas from very slowly ventilated lung units not captured by MBW_SF6_ as evidenced by the relationship between trapped gas, number of breaths and difference in LCI between systems. However, this will also increase washout time in subjects with uneven ventilation distribution as it will take longer to clear endogenous tracer gas from their lungs compared to SF_6_, which may not equilibrate in extremely slowly ventilated lung units.

FRC measured by MBW is subject to the same limitations as other gas dilution techniques in that only communicating lung units will contribute to measured volume, while FRC measured by body plethysmography includes all compressible gas volume. Thus, in subjects with significant peripheral airway obstruction we would expect differences between FRC_pleth_ and FRC_MBW_, and indeed FRC measured by both MBW techniques was lower than that measured by plethysmography. However, we observed that FRC_N2_ more closely agreed with FRC_pleth_. These results suggest that the difference between FRC_pleth_ and FRC_SF6_ may reflect trapped gas volume and that the volume contribution of slowly ventilated lung regions, not captured during MBW_SF6_, results in lower FRC_SF6_ values. Consequently, during MBW_N2_ subjects with CF required significantly more breaths to complete washout leading to the disproportionately higher CEV_N2_ compared to CEV_SF6_. Our data demonstrate that these differences are progressively more pronounced with worsening obstructive lung disease. LCI_N2_ was shown to increase disproportionately more than LCI_SF6_ with greater disease severity (increased FRC_pleth_ and lower FEV_1_) and as such may be able to more accurately reflect the degree of VI than LCI_SF6_.

These interpretations are based on the assumption that the additional gas volume measured during MBW_N2_ can be attributed to measurement of gas in extremely slowly ventilated lung units. However, a further unquantifiable amount of tissue dissolved N_2_ will diffuse from the blood into the alveoli during MBW_N2_, particularly during long washouts seen in subjects with significant VI. Most evidence would suggest unless lung disease is severe the tissue N_2_ contribution will be relatively low.[19] The close correspondence of FRC_N2_ and FRC_pleth_ observed in this study would support this hypothesis.

While it would appear that MBW_N2_ is better able to reflect the degree of peripheral airway disease than MBW_SF6_, washout times will be substantially longer in subjects with significant VI. Long washout times may limit the feasibility of MBW_N2_ in the clinical setting. This limitation could potentially be overcome by choosing higher cut-off concentrations earlier in the washout. Preliminary evidence [27] would suggest that this is possible without compromising the sensitivity of MBW_N2_. Investigation into the minimal number of trials required to achieve reproducible results; another option to shorten the test duration, is ongoing.

In conclusion, MBW_N2_ offers a valid tool to investigate obstructive lung disease in CF. Furthermore, future studies in younger patients are required to better understand the sensitivity of multiple breath N_2_ washout in this age group. In addition, interventional studies similar to those performed with MBW_SF6_ are needed to further clarify the role of MBW_N2_ as an outcome measure in clinical trials in CF patients.
